# Temporomandibular disorders among medical students in China: prevalence, biological and psychological risk factors

**DOI:** 10.1186/s12903-021-01916-2

**Published:** 2021-10-26

**Authors:** Jing Wu, Zelun Huang, Yong Chen, Yifan Chen, Zhongqiang Pan, Yu Gu

**Affiliations:** 1 Department of Stomatology, Huzhou Traditional Chinese Medicine Hospital, Zhejiang Chinese Medicinal University, Huzhou, Zhejiang China; 2 Guangzhou Key Laboratory of Basic and Applied Research of Oral Regenerative Medicine, Affiliated Stomatology Hospital of Guangzhou Medical University, Guangzhou, Guangdong China; 3 Department of Stomatology, Stomatological Hospital of Honghuagang, Zunyi, Guizhou China; 4 Department of Acupuncture, Huzhou Traditional Chinese Medicine Hospital, Zhejiang Chinese Medicinal University, Huzhou, Zhejiang China; 5 Department of Stomatology, Zhuhai Campus of Zunyi Medical University, 368 Jinwan Road, Zhuhai, 519041 Guangdong Province China

**Keywords:** Temporomandibular disorders, Psychological factors, Parafunctional habits

## Abstract

**Background:**

The purpose of the present study is to evaluate the prevalence of temporomandibular disorders (TMD) and their associated biological and psychological factors in Chinese university students.

**Methods:**

A total of 754 students were included from Zunyi Medical University, each participant completed questionnaires and clinical examinations according to the Diagnostic Criteria for Temporomandibular Disorders.

**Results:**

The overall prevalence of TMD was 31.7% among medical students. Subjects with TMD had a high prevalence of bruxism, empty chewing, unilateral chewing, chewing gum, anterior teeth overbite, anterior teeth overjet, depression, anxiety, and sleep disturbance. Moreover, sleep bruxism, empty chewing, unilateral chewing, anterior teeth overbite, depression, and anxiety were the strongest risk factors for TMD.

**Conclusions:**

Individuals with TMD have a high prevalence of psychological distress and oral parafunctional habits. Except for the psychological factors associated with TMD, bruxism, abnormal chewing, and malocclusion also shared similar risks for TMD.

**Supplementary Information:**

The online version contains supplementary material available at 10.1186/s12903-021-01916-2.

## Introduction

Temporomandibular disorders (TMD) are common clinical musculoskeletal pain syndromes involving the surrounding musculature and temporomandibular joint, mainly characterized by limited joint movement, articular sounds, or joint and muscular pain [1]. The prevalence of TMD is quite variable from 7 to 30%, and symptoms of TMD are common among adults aged 20–40 years old [2, 3]. When TMD manifests as severe clicking, pain, or limited mobility of joints, it consistently affects the quality of life and forces patients to seek help. Due to the high prevalence and high cure rates of TMD, it is important for TMD to identify TMD patients and pay attention to complex etiologies.

The etiology and pathophysiology of TMD have been considered to be multifactorial and complex, consisting of trauma, stress, parafunctional habits, as well as psychological, hereditary, and occlusal factors [3, 4]. The most acceptable theory of TMD etiology is the biopsychological-based model and the combination of initiating and persistent factors interference with enhancing TMD progression [5–7]. Parafunctional habits, such as bruxism and clenching, are biological etiology factors for symptoms observed commonly in TMD  [8–10]. In most cases, parafunctional habits mediating the increased tension of the masticatory musculature are the reason for TMD symptoms, and persistent parafunction contributes to worsening TMD symptoms. And associations between malocclusions and clinical signs of TMD were also detected in several studies, particularly, TMDs have associations with functional occlusion factors [11, 12]. Furthermore, TMD patients have heightened levels of depression, anxiety, and stress, as well as somatic awareness dysfunction, and diverse psychological factors have been considered as potential risk factors for the development of TMD [13–15]. In addition, sleep disturbance, including insomnia, sleep disruption, or insufficiency, is found to be a possibly associated factor for TMD [16, 17]. Comorbidities of psychologic distress and sleep disturbance are not uncommon in TMD.

Based on the diverse and complex etiology of TMD, the identification of TMD symptoms as well as associated factors would contribute to promoting abilities to detect the earlier stage of TMD, and early interventions ameliorate the TMD pain and improve the quality of life. Therefore, this study was aimed to determine the prevalence of TMD and its association with biological and psychological factors in medical university students.

## Methods

### Participants

This research was conducted in the Zhuhai campus of Zunyi Medical University, students were selected by simple random sampling. The inclusion criteria were as follows: (1) The dentition belongs to the permanent dentition, and the second molar is fully erupted; (2) No history of orthodontic treatment; (3) Volunteer to participate in research, and cooperate with completing a series of questionnaires and clinical examination. And the exclusion criteria were as follows: (1) Diagnosis of other orofacial pain disorders, polyarthritis, and other rheumatoid disease; (2) Diagnosis of neurologic and neuropsychiatric diseases. Each participant was required to complete a series of questionnaires, including oral behaviors checklist, TMD symptom questionnaire, psychological questionnaire, and sleep status questionnaire. In addition, each participant completed a clinical examination according to the guidelines of the Diagnostic Criteria for TMD (DC/TMD) to identify participants having TMD [18]. A total of 754 participates were included in the current study and written informed consent was obtained from all subjects. This study was approved by the ethical committee of the Zunyi Medical University (2019-H007).

### Assessment of TMD

Oral behaviors were gathered in detail through the questionnaire, containing sleep bruxism, awake bruxism, empty chewing, unilateral chewing, and chewing gum [18]. The TMD symptom questionnaire and clinical examination were conducted based on the guidelines of the DC/TMD [18]. The symptom questionnaire of the DC/TMD involves 14 items relating to characteristics of TMD symptoms (specifically facial pain, headaches, temporomandibular joint sounds, temporomandibular joint closed and open locking) and is designed to gather necessary information for deriving the DC/TMD Axis I diagnosis (Additional file 1). Participants are involved in a protocolised clinical evaluation by trained dental specialists according to the clinical examination form in DC/TMD. TMD diagnoses are subsequently made based on the symptom questionnaire, clinical examination form and the DC/TMD diagnostic algorithms. And according to the TMD diagnosis, patients were divided into three groups: the pain-related TMD, the intra-articular TMD, and the combined TMD.

### Assessment of psychological factors

The psychological questionnaires comprised the Patient Health Questionnaire-9 (PHQ-9) and the Generalized Anxiety Disorder Scale-7 (GAD-7), while sleep disturbance was evaluated by the Pittsburgh Sleep Quality Index (PSQI). The PHQ-9 has been adopted widely as a screening and diagnostic tool for the depressive disorder [19]. Depression was classified into five grades according to the computed scores: normal (0–4 scores), mild (5–9 scores), moderate (10–14 scores), severe (15–19 scores), and extremely severe (20–27 scores). The GAD-7 has composite reliability as well as criterion-related validity for assessing generalized anxiety disorder [20]. Anxiety was also classified into five grades: normal (0–4 scores), mild (5–9 scores), moderate (10–13 scores), severe (14–18 scores), and extremely severe (19–21 scores). The PSQI was a self-rating sleep quality scale developed by Buysse et al. [21], which consisted of seven indicators, including sleep quality, sleep latency, sleep duration, habitual sleep efficiency, sleep disturbance, use of sleep medicine, and daytime dysfunction. Each indicator was scored on a scale of 0–3, and the cumulative score of each indicator was the total score of PSQI, which ranged from 0 to 21. The higher scores represented the worse sleep quality.

### Statistical analysis

All data were conducted using SPSS software version 22. The prevalence data were presented as percentages. The chi-square test and the independent-samples t-test were used to compare the prevalence among participates with or without TMD. Correlations between TMD and biological and psychological characteristics were assessed using the spearman test. The logistic regression analysis was used to determine possible risk factors for TMD. *p* < 0.05 was considered to be statistically significant.

## Results

### Prevalence of TMD

**Fig. 1:**
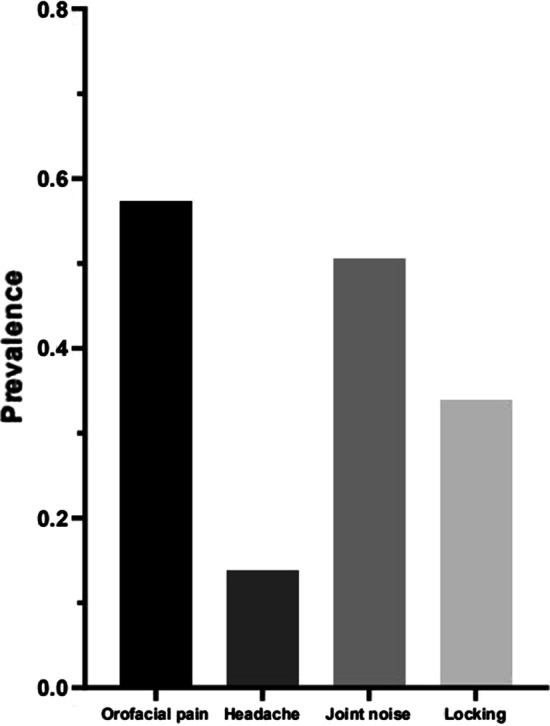
Prevalence of temporomandibular disorder symptoms in the total participants

A total of 754 students, who completed the questionnaire and the clinical examination form, were included in this study. Of the 754 students, there were 354 males and 400 females, aged 19 (18, 19) years. The overall prevalence of TMD was 31.7%, showing no significant gender difference. Orofacial pain and joint noise were the most common symptoms of TMD with the incidence of 57.3% and 50.6%, while headache and locking had the low incidence of 13.8% and 33.9% (Fig. 1). Moreover, the participants were divided into three groups as the pain-related TMD, the intra-articular TMD, and the combined TMD, with the respective prevalence of 15.5%, 66.9%, and 17.6%.

### Biological and psychological characteristics of TMD

As shown in Table 1, students with TMD had a high prevalence of sleep bruxism, awake bruxism, empty chewing, unilateral chewing, and chewing gum, compared to those without TMD. As for occlusions, a significantly higher prevalence of anterior teeth overbite and anterior teeth overjet was observed in students with TMD. In the psychological aspect, a significantly higher prevalence of depression, anxiety, and sleep disturbance was observed in patients with TMD.

**Table 1:** Comparison of biological and psychological factors among participants without or with temporomandibular disorders (TMD)

Characteristic	Participants without TMD (n = 515)	Participants with TMD (n = 239)	*p* value
*Biological factor*			
Age	18 (18, 19)	19 (18, 19)	0.172
Female	281 (54.6)	119 (49.8)	0.222
Sleep bruxism	56 (10.9)	56 (23.4)	< 0.001
Awake bruxism	16 (3.1)	18 (7.5)	0.006
Empty chewing	39 (7.6)	50 (20.9)	< 0.001
Unilateral chewing	172 (33.4)	135 (56.5)	< 0.001
Chewing gum	135 (26.2)	81 (33.9)	0.030
*Static occlusion*			
Angle’s malocclusion	185 (35.9)	92 (38.4)	0.464
Anterior teeth overbite	213 (41.3)	139 (58.1)	< 0.001
Anterior teeth overjet	163 (31.7)	102 (42.7)	0.003
Posterior teeth crossbite	10 (1.9)	2 (0.8)	0.357
Posterior scissor bite	18 (3.5)	14 (5.9)	0.173
*Psychological factor*			
Depression			
Prevalence	232 (45.0)	167 (69.9)	< 0.001
Average score	4 (3, 7)	7 (4,9)	< 0.001
Anxiety			
Prevalence	163 (31.7)	166 (69.5)	< 0.001
Average score	3 (1, 5)	6 (4, 7)	< 0.001
Sleep disturbance			
Prevalence	159 (30.9)	123 (51.5)	< 0.001
Average score	5 (3, 6)	6 (4,7)	< 0.001

Furthermore, the association between TMD and biological and psychological characteristics was analyzed (Table 2). Students with TMD had a significant correlation with sleep bruxism, awake bruxism, empty chewing, unilateral chewing, chewing gum. And in occlusion aspects, anterior teeth overbite and anterior teeth overjet were correlated with TMD. Moreover, a significant correlation was also noted between TMD and depression, anxiety, and sleep disturbance. In addition, the influence of biological and psychological factors on different types of TMDs was verified. As shown in Table 3, there were 37, 160, and 42 people in the study with groups of the pain-related TMD, the intra-articular TMD, and the combined TMD, respectively. In the pain-related TMD group, it was only positively associated with sleep bruxism, unilateral chewing, anterior teeth overbite, depression, and anxiety. In the intra-articular TMD group, it was positive correlated with gender, sleep bruxism, awake bruxism, empty chewing, unilateral chewing, anterior teeth overbite, anterior teeth overjet, depression, anxiety, and sleep disturbance. There were also significant positive correlations between combined TMD and gender, sleep bruxism, empty chewing, unilateral chewing, depression, anxiety, and sleep disturbance.

**Table 2:** Variables and risk factors for temporomandibular disorders (TMD)

Biological factor	*r*	*p* value	Static occlusion	*r*	*p* value	Psychological factor	*r*	*p* value
Age	0.050	0.172	Angle’s malocclusion	− 0.061	0.320	Depression	0.231	< 0.001
Gender	0.044	0.222	Anterior teeth overbite	0.166	< 0.001	Anxiety	0.355	< 0.001
Sleep bruxism	0.164	< 0.001	Anterior teeth overjet	0.110	0.002	Sleep disturbance	0.184	< 0.001
Awake bruxism	0.099	0.006	Posterior teeth crossbite	− 0.041	0.260			
Empty chewing	0.192	< 0.001	Posterior scissor bite	0.055	0.135			
Unilateral chewing	0.219	< 0.001						
Chewing gum	0.079	0.030

**Table 3:** Correlations of biological and psychological factors among different classifications of temporomandibular disorders (TMD)

	Pain-related TMD (n = 37)		Intra-articular TMD (n = 160)		Combined TMD (n = 42)	
	*r*	*p* value	*r*	*p* value	*r*	*p* value
*Biological factor*						
Age	0.013	0.711	0.020	0.585	0.062	0.091
Gender	0.008	0.832	0.097	0.008	0.089	0.014
Sleep bruxism	0.078	0.033	0.075	0.039	0.126	0.001
Awake bruxism	0.039	0.280	0.075	0.040	0.031	0.390
Empty chewing	0.031	0.394	0.112	0.002	0.162	< 0.001
Unilateral chewing	0.074	0.042	0.118	0.001	0.164	< 0.001
Chewing gum	0.033	0.371	0.037	0.310	0.064	0.081
*Static occlusion*						
Angle’s malocclusion	0.030	0.567	0.066	0.078	0.051	0.764
Anterior teeth overbite	0.099	0.006	0.096	0.008	0.071	0.050
Anterior teeth overjet	0.060	0.097	0.078	0.031	0.027	0.451
Posterior teeth crossbite	0.020	0.580	-0.066	0.070	0.015	0.674
Posterior scissor bite	0.013	0.720	0.003	0.926	0.092	0.051
*Psychological factor*						
Depression	0.116	0.001	0.119	0.001	0.148	< 0.001
Anxiety	0.134	< 0.001	0.230	< 0.001	0.148	< 0.001
Sleep disturbance	0.026	0.477	0.119	0.001	0.114	0.002

**Fig. 2:**
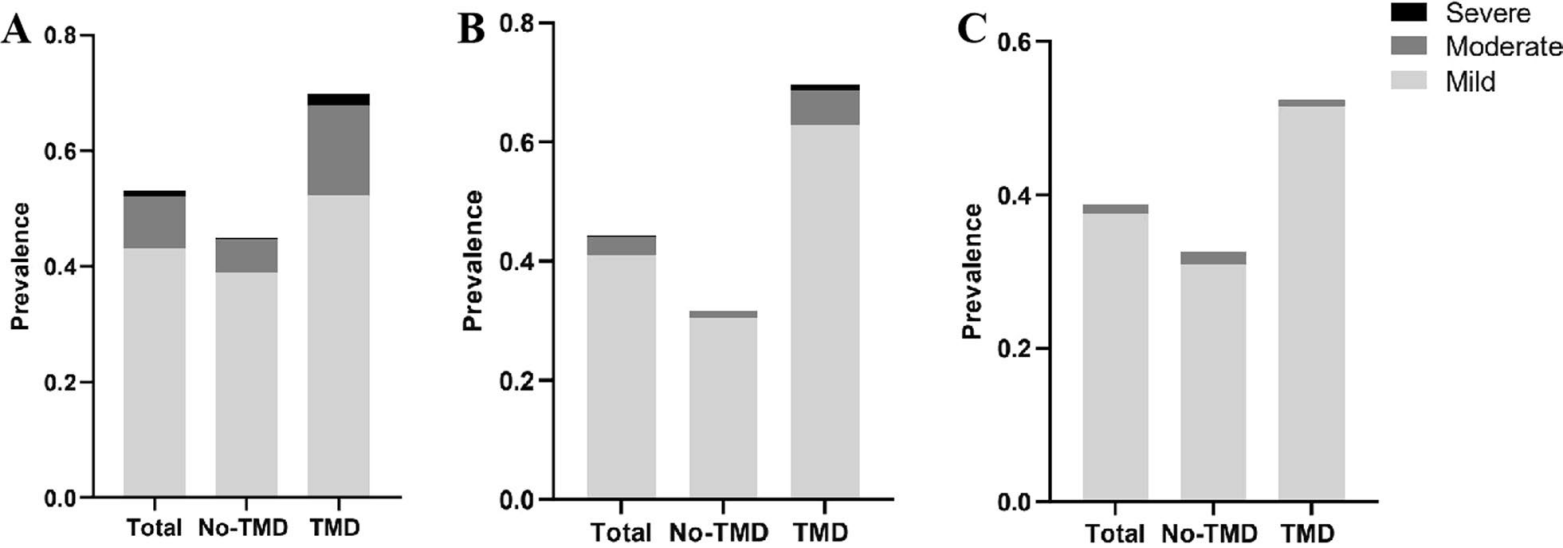
Prevalence of psychological distress and sleep disturbance. Percentage of subjects with mild, moderate, and severe distress as shown, including depression (**A**), anxiety (**B**), and sleep disturbance (**C**)

In addition, depression, anxiety, and sleep disturbance were divided as mild to severe according to PHQ-9, GAD-7, and PSQI, respectively (Fig. 2). There are 52.3% students with mild depression, 15.5% students with moderate depression, and 2.1% students with moderate-severe depression among the TMD group, which revealed significantly higher severity of depression in patients with TMD. The anxiety and sleep disturbance parameters showed a similar trend.

### Assessment of possible risk factors among TMD

In order to find out the factors as independent factors of TMD, logistic regression analyses were performed. The univariate logistic regression showed that sleep bruxism, awake bruxism, empty chewing, unilateral chewing, chewing gum, anterior teeth overbite, anterior teeth overjet, depression, anxiety, and sleep disturbance were risk factors for TMD. According to the multiple logistic regression model, sleep bruxism, empty chewing, unilateral chewing, anterior teeth overbite, depression, and anxiety were independent risk factors for TMD (Table 4).

**Table 4:** Univariate and multivariate logistic regression models of risk factors for temporomandibular disorders

	Univariable		Multivariable	
	HR (95 %CI)	*p* value	HR (95 %CI)	*p* value
*Biological factor*				
Gender	1.211 (0.891, 1.646)	0.222		
Sleep bruxism	2.508 (1.668, 3.772)	< 0.001	1.943 (1.217, 3.104)	0.005
Awake bruxism	2.540 (1.272, 5.073)	0.008	1.284 (0.567, 2.908)	0.548
Empty chewing	3.229 (2.056, 5.071)	< 0.001	2.489 (1.484, 4.173)	0.001
Unilateral chewing	2.589 (1.890, 3.545)	< 0.001	1.679 (1.178, 2.393)	0.004
Chewing gum	1.443 (1.035, 2.001)	0.030	0.977 (0.668, 1.429)	0.906
*Static occlusion*				
Angle’s malocclusion	1.276 (0.785, 3.461)	0.364		
Anterior teeth overbite	1.234 (1.115, 1.367)	< 0.001	1.193 (1.014, 1.403)	0.033
Anterior teeth overjet	1.262 (1.073, 1.485)	0.005	1.017 (0.786, 1.317)	0.895
Posterior teeth crossbite	0.426 (0.093, 1.960)	0.273		
Posterior scissor bite	1.718 (0.840, 3.515)	0.138		
*Psychological factor*				
Depression	2.829 (2.042, 3.921)	< 0.001	1.656 (1.143, 2.400)	0.008
Anxiety	4.911 (3.523, 6.844)	< 0.001	3.351 (2.325, 4.829)	< 0.001
Sleep disturbance	2.021 (1.505, 2.714)	< 0.001	1.324 (0.946, 1.852)	0.101

*h* hazard ratio, *CI* confidence interval

## Discussion

Academic stress is extremely high among medical university students in China due to educational standards, personal desires, and societal demands, which leads to psychological distress and sleep dysfunction and then contributes to TMD. Moreover, psychological distress also contributes to parafunctional habits like bruxism and abnormal chewing, which may associate with TMD. Thus, the current study aimed to investigate the effects of psychological aspects, parafunctional habits and malocclusions on TMD among medical university students in China.

In the present study, 31.7% of participants had at least one TMD symptom, which was considerably higher than the prevalence of TMD (only 10–20%) in Asian youngers in the past century [22, 23]. It is worthy to further investigate the reasons resulting in a substantial increase in TMD prevalence among participants. One of the main findings in the present study was that psychological distress was more common in participants with TMD than those without TMD, of which depression, anxiety, and sleep disturbance in TMD patients were present in 69.9%, 69.5%, and 51.5%, respectively. Moreover, depression and anxiety showed strong significant associations with TMD, which were independent risk factors for TMD. Similar results were reported by Anna and colleagues, a higher prevalence of depression, anxiety, and somatization among students with TMD symptoms in Poznań university of medical sciences [13]. Another research indicated that PHQ-9 and GAD-7 questionnaires for screening depression and anxiety should be considered in the diagnosis of TMD patients, which supported the present findings [15]. A possible explanation for associations between psychological diseases and TMD would be due to that psychological pressure leads to grinding and clenching, which exacerbates the masticatory muscle tension contributing to TMD [24–26]. Another major finding was that sleep disturbance was also more common in TMD patients, while it was only associated with intra-articular TMD. In another Chinese adolescent cohort research, a high prevalence of sleep disturbance and TMD symptoms were presented in senior high students, and sleep disturbance increased the risk of TMD symptoms [27]. Rener-Sitar and colleagues reported that TMD patients with pain possessed much poorer sleep quality, which indicated that sleep quality should be evaluated in TMD patients especially those with pain [28]. A circular relationship of mutual deleterious influences between TMD symptoms and sleep exists, thus sleep improvement should be targeted for negative effects on TMD.

Additionally, TMD symptoms were assessed concerning the presence of parafunctional habits including bruxism and abnormal chewing. The present study showed that subjects with TMD possessed a higher prevalence of certain parafunctional habits such as bruxism, empty chewing, unilateral chewing, and chewing gum, while sleep bruxism, empty chewing, and unilateral chewing were independent risks of developing TMD. A previous study also showed that oral parafunctional habits were commonly observed in college preparatory students, which were considered contributory factors for TMD symptoms [29]. Furthermore, TMD was more prevalent in cases with bruxism, while bruxism induced overloading of the temporomandibular joint due to degenerative changes [30]. In a survey of Turkish university students, unilateral chewing was significantly associated with TMD, which disturbed the rhythmic coordination of the jaw and neck muscles during chewing contributing to TMD symptoms [31]. Interestingly, those participating in the previous study with longer chewing cycles and length had more prevalence of experiencing TMD, which was consistent with the current findings [32]. It was explained that oral parafunctional habits might cause TMD symptoms due to the overloading of musculoskeletal structures. The relationship between TMD and occlusion is still controversial, and although some professionals considered that malocclusion is one of the factors for initiation of TMD. In the present study, TMD possessed a higher prevalence of anterior teeth overbite and anterior teeth overjet, which were independent risks of developing TMD. Thilander groups observed that TMDs were significantly associated with posterior crossbite, and suggested that malocclusions should be treated orthodontically in an early age to protect against TMD [33]. In contrast, Al-Ani and Špalj et al. found that symptoms of TMDs seemed to be poorly related to malocclusions [34, 35]. Although the evidence available does not allow to establish a unambiguous relationship between malocclusions and TMD, the effects of malocclusions in growing patients of TMDs deserve further investigation.

Apart from the noteworthy findings, there were some potential limitations in the present study. First, all participants were only from one medical university, the extended research incorporating students from other universities should be planned to increase sample size and truly reflect the situation of TMD in medical students. Second, the current study had not evaluated the imaging datas, which were mainly assessed through X-ray or magnetic resonance imaging. It was difficult to comprehensively and accurately evaluate TMD conditions due to the lack of the imaging studies. Finally, the present study was a cross-sectional research, future studies should be considered a longitudinal study design to confirm these factors as the predictors to TMD (Additional file 1).

## Conclusions

A high prevalence of TMD symptoms was reported among Chinese students at the university of medical sciences. TMD in those population was considered as multifactorial condition, in which psychological distress symptoms as depression, anxiety as well as sleep disturbance correlated with TMD. Some oral parafunctional habits and malocclusions showed association with TMD, mainly the presence of bruxism and abnormal chewing were potential risk factors to TMD.

## Supplementary Information

**Additional file 1.** TMD Symptom Questionnaire.

## Data Availability

The datasets used and/or analysed during the current study are available from the corresponding author on reasonable request.
