# Supplementary material for: Temporomandibular disorders among medical students in China: prevalence, biological and psychological risk factors
Source: BMC Oral Health. 2021 Oct 26;21:549. doi: 10.1186/s12903-021-01916-2 (PMC8549286; doi:10.1186/s12903-021-01916-2)
Supplement: Supplementary file 1 — Additional file 1. TMD Symptom Questionnaire. [file 12903_2021_1916_MOESM1_ESM.doc]

**TMD Symptom Questionnaire**

1. Have you ever had pain in your jaw, temple, in the ear, or in front of the ear on either side?

**If you answered NO, then skip to Question 5.**

1. How many years or months ago did your pain in the jaw, temple, in the ear, or in front of the ear first begin?

_______years ______months

1. In the last 30 days, which of the following best describes any pain in your jaw, temple, in the ear, or in front of the ear on either side? Select ONE response.

No pain

Pain comes and goes

Pain is always present

**If you answered NO to Question 3, then skip to Question 5.**

1. In the last 30 days, did the following activities change any pain (that is, make it better or make it worse) in your jaw, temple, in the ear, or in front of the ear on either side? (YES or NO)
2. Chewing hard or tough food
3. Opening your mouth, or moving your jaw forward or to the side
4. Jaw habits such as holding teeth together, clenching/grinding teeth, or chewing gum
5. Other jaw activities such as talking, kissing, or yawning
6. In the last 30 days, have you had any headaches that included the temple areas of your head?

**If you answered NO to Question 5, then skip to Question 8.**

1. How many years or months ago did your temple headache first begin?

_______years ______months

1. In the last 30 days, did the following activities change any headache (that is, make it better or make it worse) in your temple area on either side? (YES or NO)
2. Chewing hard or tough food
3. Opening your mouth, or moving your jaw forward or to the side
4. Jaw habits such as holding teeth together, clenching/grinding, or chewing gum
5. Other jaw activities such as talking, kissing, or yawning
6. In the last 30 days, have you had any jaw joint noise(s) when you moved or used your jaw?
7. Have you ever had your jaw lock or catch, even for a moment, so that it would not open ALL THE WAY?

**If you answered NO to Question 9 then skip to Question 13.**

1. Was your jaw lock or catch severe enough to limit your jaw opening and interfere with your ability to eat?
2. In the last 30 days, did your jaw lock so you could not open ALL THE WAY, even for a moment, and then unlock so you could open ALL THE WAY?

**If you answered NO to Question 11 then skip to Question 13.**

1. Is your jaw currently locked or limited so that your jaw will not open ALL THE WAY?
2. In the last 30 days, when you opened your mouth wide, did your jaw lock or catch even for a moment such that you could not close it from this wide open position?

**If you answered NO to Question 13 then you are finished.**

14. In the last 30 days, when you jaw locked or caught wide open, did you have to do something to get it to close including resting, moving, pushing, or maneuvering it?
